# Survival and Axonal Outgrowth of the Mauthner Cell Following Spinal Cord Crush Does Not Drive Post-injury Startle Responses

**DOI:** 10.3389/fcell.2021.744191

**Published:** 2021-11-19

**Authors:** Steven J. Zottoli, Donald S. Faber, John Hering, Ann C. Dannhauer, Susan Northen

**Affiliations:** ^1^Department of Biology, Williams College, Williamstown, MA, United States; ^2^Eugene Bell Center for Regenerative Biology and Tissue Engineering, Marine Biological Laboratory, Woods Hole, MA, United States; ^3^Albert Einstein College of Medicine, Rose F. Kennedy Center, Bronx, NY, United States

**Keywords:** spinal cord regeneration, functional recovery, startle responses, Mauthner cells, adult goldfish

## Abstract

A pair of Mauthner cells (M-cells) can be found in the hindbrain of most teleost fish, as well as amphibians and lamprey. The axons of these reticulospinal neurons cross the midline and synapse on interneurons and motoneurons as they descend the length of the spinal cord. The M-cell initiates fast C-type startle responses (fast C-starts) in goldfish and zebrafish triggered by abrupt acoustic/vibratory stimuli. Starting about 70 days after whole spinal cord crush, less robust startle responses with longer latencies manifest in adult goldfish, *Carassius auratus.* The morphological and electrophysiological identifiability of the M-cell provides a unique opportunity to study cellular responses to spinal cord injury and the relation of axonal regrowth to a defined behavior. After spinal cord crush at the spinomedullary junction about one-third of the damaged M-axons of adult goldfish send at least one sprout past the wound site between 56 and 85 days postoperatively. These caudally projecting sprouts follow a more lateral trajectory relative to their position in the fasciculus longitudinalis medialis of control fish. Other sprouts, some from the same axon, follow aberrant pathways that include rostral projections, reversal of direction, midline crossings, neuromas, and projection out the first ventral root. Stimulating M-axons in goldfish that had post-injury startle behavior between 198 and 468 days postoperatively resulted in no or minimal EMG activity in trunk and tail musculature as compared to control fish. Although M-cells can survive for at least 468 day (∼1.3 years) after spinal cord crush, maintain regrowth, and elicit putative trunk EMG responses, the cell does not appear to play a substantive role in the emergence of acoustic/vibratory-triggered responses. We speculate that aberrant pathway choice of this neuron may limit its role in the recovery of behavior and discuss structural and functional properties of alternative candidate neurons that may render them more supportive of post-injury startle behavior.

## Introduction

The regenerative capacity of the spinal cord in anamniotes is well documented. Lamprey, teleost fish, and amphibians have the ability to regain locomotory movements after spinal cord injury (reviewed by Windle, 1956; Cohen et al., 1988; Larner et al., 1995; Bernhardt, 1999; Becker and Becker, 2008, 2014; Diaz Quiroz and Echeverri, 2013; Vajn et al., 2013; Zupanc and Sîrbulescu, 2013; Bloom, 2014; Rodemer et al., 2020; Haspel et al., 2021; Van houcke et al., 2021). Surprisingly, the recovery of other motor control behaviors such as equilibrium, feeding, and startle responses has been less well studied. A pair of identifiable cells, the Mauthner cells (M-cells), are known to initiate fast C-type startle responses (fast C-starts; Zottoli, 1977; Eaton et al., 1981; Hecker et al., 2020b) and S-starts (Liu and Hale, 2017). Axonal regrowth of lesioned Mauthner axons (M-axons) results in functional synaptic connections with motoneurons in *Xenopus* tadpoles (Lee, 1982) and recovery of fast C-starts in larval zebrafish (Bhatt et al., 2004; Hecker et al., 2020a). However, the role of M-cells in post-injury startle responses of adult fish is less clear. Startle responses can be elicited by abrupt acoustic/vibratory stimuli in adult goldfish, *Carassius auratus*, after spinal cord crush. These responses are less frequent with long latencies and are less robust compared to those in sham-operated control fish (Zottoli and Freemer, 2003). M-axons in adult fish have been shown to traverse a spinal cord wound in some studies (Becker et al., 1998; Becker and Becker, 2001) but not in others (Sharma et al., 1993; Becker et al., 1997). Intracellular labeling of M-axons in the adult goldfish damaged by spinal cord crush initiate sprouting in a few days (Koganti et al., 2020) but choose aberrant pathways between 30 and 42 days postoperatively (Zottoli et al., 1994; Zottoli and Faber, 2000). Little is known whether these sprouts are maintained or re-routed and form functional synapses over long post-operative intervals. We report here the results of experiments in adult goldfish with the aim of distinguishing behavioral, morphological, and electrophysiological consequences of M-cell axotomy by spinal cord crush. We ask whether: (1) M-axon sprouts traverse a crush wound at the junction of the medulla oblongata and spinal cord (spinomedullary level, SML), (2) M-axon regrowth contributes to post-injury startle responses, (3) M-cells can survive long postoperative intervals and maintain axon sprouts, and (4) post-injury startle responses in fish with an SML-crush are the same or differ from responses after a combination of M-cell ablation and SML-crush. We found that some M-axon sprouts project caudally, crossing the wound site after SML-crush, but they do not play a substantive role in post-injury startle responses. The similarity in such responses after SML-crush with and without M-cells supports these results. We assess possible mechanisms that may limit the ability of the M-cell to participate in behavioral recovery. Results such as these highlight the need to employ multidisciplinary approaches to expose the interactions that define complex neural circuit functions and their disturbances.

## Materials and Methods

### Terminology

An abrupt acoustic/vibratory stimulus elicits Mauthner cell-initiated fast C-starts in adult goldfish (Zottoli, 1977; Eaton et al., 1981; Zottoli et al., 1999). An alternate neuronal pathway that is capable of initiating C-type startle responses, albeit with longer latencies than M-cell-initiated responses, was revealed after lesioning of the M-cell initial segment and soma (Eaton et al., 1982; Zottoli et al., 1999). Startle responses that return after a crush wound at the spinomedullary level (SML) are elicited less frequently, are less robust, and have a longer latency from stimulus onset to movement. This emergent behavior has been described as a “recovered C-start” (Zottoli and Freemer, 2003). This terminology is misleading since the neuronal circuitry for such startle behavior is not known at this time, and, therefore, we will use the term “post-injury startle responses,” based on their timing and intrinsic dynamics.

### Fish Care

Common goldfish, *Carassius auratus*, (purchased in the fall from Hunting Creek Fisheries Inc., Thurmont, MD, United States), of 10.2 ± 1.2 cm standard length (mean ± SD range; 9–15 cm) were allowed to acclimate for a minimum of 2–3 weeks prior to use. Fish were housed individually in 23 cm × 17 cm × 14 cm deep tanks with 4 L of conditioned water (NovAqua; Kordon, Inc.). The water was continuously aerated and the mean temperature was 22.3 ± 1°C; (mean ± SD; range = 17.5–23.7°C). The fish were exposed to an alternating 12-h light, 12-h dark cycle. They were fed Hikari Staple food (mini pellet, floating type, Kyorin Food Ind. Ltd.) three times a week followed 2 h later by cleaning the tank and replacing the water with fresh, conditioned tap water. The fish were breeder stock between 6 months and 1.5 years old (Hunting Creek Fisheries, Inc., personal communication). The care and treatment of fish was in compliance with the Guide for the Care and Use of Laboratory Animals and was approved by the Williams College IACUC.

### Brain Dissection

**FIGURE 1:**
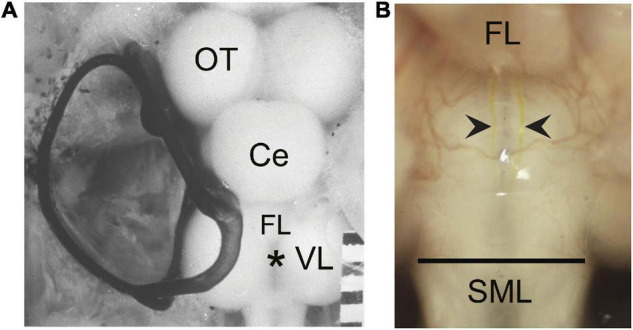
The wound site and visual identification of Mauthner axons (M-axons). **(A)** Goldfish brain with the left set of semicircular canals filled with India ink. An asterisk marks the location enlarged in panel **(B)**. Distance between calibration marks = 1 mm. Ce, cerebellum; OT, optic tectum; FL, facial lobe; VL, vagal lobe. **(B)** Goldfish hindbrain in which the medulla oblongata has been exposed (see section “Materials and Methods”). Wound site is at the caudal border of the vagal lobes near the junction of the medulla oblongata and spinal cord (spinomedullary level, SML). The M-axon comes within 100 μm of the surface of the medulla oblongata and is visible between the vagal lobes with the aid of a dissecting microscope. As a result, the M-axons can be reliably penetrated for recording, stimulation, and dye injection. Successful Lucifer yellow fills of M-axons are indicated by arrowheads just caudal to the facial lobe (FL). The dye can be seen with tungsten light. The diameter of the spinal cord just caudal to the vagal lobes is about 2 mm. The orientation of the brain is with rostral at the top and caudal at the bottom in this and all subsequent figures.

Fish were initially anesthetized in 0.024% ethyl-m-aminobenzoate (Sigma-Aldrich Co., St. Louis, MI, United States) until breathing ceased and were then transferred to an operating chamber where chilled water containing 0.01% of anesthetic was recirculated through the mouth and over the gills (under these conditions, the temperature of water measured in the opercular cavity stabilized at 10°C). A goldfish brain with the left semicircular canals filled with India ink is shown in Figure 1A. The area extending from the posterior margin of the corpus cerebellum (Ce) caudally to the spinal cord was exposed. Overlying muscle, cartilage, and fat were removed to expose the vagal lobes and rostral spinal cord at the site of the asterisk in Figure 1A.

### Spinomedullary Whole Spinal Cord Crush Technique

The tips of a No. 5 Dumont forceps were separated, placed on either side of the spinal cord with the aid of a dissecting microscope, and lowered until they touched the floor of the brain case. The forceps were moved rostrally to the caudal edge of the vagal lobes, a site that is at the junction of the spinal cord and medulla oblongata. The tips were oriented perpendicular to the spinal cord axis at this spinomedullary level (SML; Figure 1B) and were then closed tightly and held together for 1–2 s. The anesthetized fish moved slightly, giving a preliminary indication that the medullary tissue had been damaged. After this initial crush, the tips were again closed and held for another 1–2 s. Although the crush did not disconnect the spinal cord from the medulla oblongata, a distinct line was evident where the crush had been made. Little bleeding resulted from this wound. We estimate that the wound site extended from the posterior edge of the vagal lobes for less than 1 mm caudally. SML-crush damages and ultimately results in separation of all descending axons (Zottoli and Freemer, 2003). Control fish had a sham-operation; the brain was exposed and forceps placed as described above but there was no crush before sealing the skull. Sham fish behaved normally after recovery from general anesthesia.

### Skull Sealing Procedure

After an SML-crush, the brain and spinal cord were protected from additional trauma and osmotic shock by filling the surgical opening with a Vaseline-paraffin oil mixture to a level just below the skull. A piece of thin plastic the size of the opening in the skull was placed on this solution. Thirty-gauge stainless steel wire was looped through two small accessory holes drilled on either side of and rostral to the operation hole. The wire’s ends were twisted together caudally where a loop was made on one of the ends. The caudal loop was anchored to musculature just behind the skull with silk suture thread. The twisted wire and string acted as a secure framework for the vinyl polysiloxane impression material (Imprint, 3 M) used to “cap” the skull. After the operation, the recirculating anesthetic solution was replaced with conditioned tap water until the fish initiated breathing in approximately 5–15 min.

### Behavior

Fish were returned to their home tanks and monitored for 30–60 min to assess the effectiveness of the operation. The sham-operated fish appeared normal on recovery from anesthetic. After SML-crush and recovery from the anesthetic, fish lay on their sides with no movement caudal to the wound. If any spontaneous movements were detected, the fish was not included in this study. Fish were fed postoperatively with presoaked mini pellets that sunk to the bottom of the tank and could be ingested by the fish. The fish were observed daily for the first 10 postoperative days to monitor carefully the effect of the operation.

The behavioral test tank was abruptly lifted to deliver an acoustic/vibratory stimulus as has been described elsewhere (Zottoli et al., 1999; Zottoli and Freemer, 2003). We used computer software (KNOWAL, Nissanov, 1991) to determine: (1) whether a post-injury startle response occurred and (2) to determine kinematic parameters including latency from stimulus onset to response, escape trajectory angle, straight-line center of mass distance 70 ms after the start, and linear velocity of the center of mass movement. In some cases responses were monitored after stimulation with two cycles of a 200 Hz sinusoidal signal generated by an underwater loudspeaker (Universal, Model UW-30). Although this stimulus is less effective in eliciting post-injury startle responses, it did not influence our results other than increasing the postoperative intervals at which the responses were elicited after SML-crush (Zottoli and Freemer, 2003).

All fish were tested preoperatively for their ability to respond to an acoustic/vibratory stimulus with a C-start. One set of six trials with an inter-trial interval of at least 2 min was given prior to SML-crush. Fish were screened during preoperative testing to meet the following three criteria: (1) each fish had to respond to the stimulus with C-starts in at least three of the six trials, (2) at least one C-start had to be to the left and one to the right, and (3) the fish silhouette had to be compatible with the software thinning algorithm (e.g., some fish had silhouettes that made it difficult for software analysis). Experimental fish were tested again 10 days postoperatively to ensure that the acoustic/vibratory stimulus did not elicit movement of trunk or tail musculature. These fish had a head-level response but no body movement at this postoperative interval. Fish were then observed weekly for return of the ability to eat food pellets from the water surface, for the return of equilibrium, and for a body response elicited by a tap on the home tank. Once post-injury startle responses occurred, fish were tested with the acoustic/vibratory stimulus as described above in blocks of six trials with a 2 min inter-trial interval. Testing for startle responses occurred at random times during the day/light cycle, and, in general, a set of six trials took approximately 1 h to complete.

### Lucifer Yellow Fill Technique

Lucifer yellow was injected into M-axons of SML-crush fish to determine the extent of M-axon regrowth. SML-crush fish were anesthetized in 0.024% ethyl-m-aminobenzoate (Sigma-Aldrich Co., St. Louis, MI, United States) until breathing ceased and were then transferred to an operating chamber where chilled water containing 0.01% of anesthetic was recirculated through the mouth and over the gills. The medulla oblongata was exposed between the vagal lobes by removing the “cap” of dental impression material, the wire, and thread that anchored it and by suctioning away the Vaseline-paraffin mixture. The choroid plexus between the vagal lobes was torn with forceps and the vagal lobes were spread and held apart with Kimwipes to expose the surface of the medulla oblongata below the fourth ventricle (Figure 1B). The tip of a microelectrode filled with Lucifer yellow (5% in distilled water; Lucifer yellow, CH lithium salt; Sigma-Aldrich) was lowered to the surface of the medulla oblongata just caudal to the facial lobe and well rostral to the SML-crush site to ensure that the microelectrode did not damage the retracted M-axon tip. The axons are visible at this level where they come within 100 μm of the medullary surface (see Zottoli et al., 1994, Figure 5). Once an axon was penetrated, as determined by a stable resting potential, it was filled iontophoretically with dye (−10 to −30 nA for 200 ms three times a second for a minimum of 30 min). The success of the injection could be judged by observing the rapid entry of dye into the M-axons with a dissecting microscope and tungsten light. Axons filled with dye are marked by the arrowheads in Figure 1B. After the dye injections were completed, the anesthetized fish were perfused through the heart with 100 mL of 10% formalin in phosphate buffer (Fisher Scientific, Inc.). The brain and rostral spinal cord were removed and placed in fresh fixative overnight. The tissue was then dehydrated, and cleared in methyl salicylate before observing the whole brain (“brain wholemount”) under the fluorescent microscope. An example of filled, uninjured M-axons in the cleared brain wholemounts can be seen in Figure 1 of Koganti et al. (2020). Occasionally other axons were inadvertently filled with dye. The sprouts of these non-M-axons were clearly distinguishable from the M-axon sprouts in wholemount brain preparations.

### Measurements of Mauthner-Axon Regrowth From Brain Wholemounts

**FIGURE 2:**
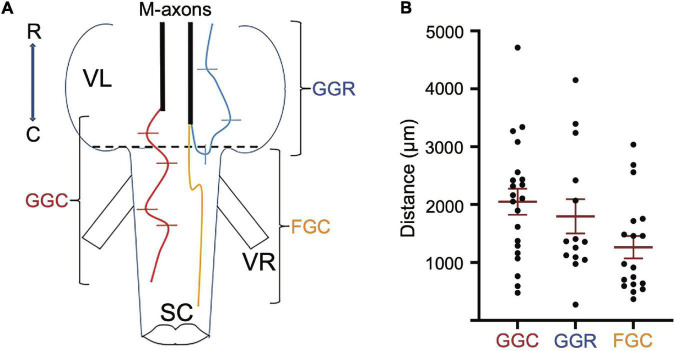
Regrowth of axotomized M-axons. **(A)** Hindbrain schematic showing three M-axon sprouts and the method of measurement. For each axon, filled with Lucifer yellow, the caudal sprout that had the greatest growth caudally (GGC, red) was measured in segments. In this example, five segments delineated by lines were spliced together to give the GGC. The rostral sprout that had the greatest growth rostrally (GGR, blue) was measured in this example by combining the length of three segments. Measurements were first made when the sprout projected rostrally. The sprout that extended the furthest caudally from the wound site (furthest growth caudally, FGC, orange) was measured as the straight line distance from the caudal edge of the vagal lobes (SML, dashed line) to the end of the sprout. VR, ventral root; VL, vagal lobe; SC, spinal cord; R, rostral; C, caudal. **(B)** Plots of values (mean ± S.E.M.) for greatest growth caudally (GGC), greatest growth rostrally (GGR), and furthest growth caudally (FGC).

Mauthner-axon sprout measurements were taken from brain wholemounts with a fluorescent microscope. The measurements for each axon include:

(a) The length of the sprout with the greatest growth rostrally (GGR). Measurements were made in segments along a meandering sprout and therefore represent the total growth. In some cases the sprout initially projected caudally and/or laterally; measurements were only made once the sprout began its rostral trajectory (Figure 2A, blue sprout).
(b) The length of the sprout with the greatest growth caudally (GGC). Measurements were made in segments along a sprout and therefore represent the total caudal growth. In some cases the sprout initially projected rostrally and/or laterally; measurements were only made once the sprout began its caudal trajectory (Figure 2A, red sprout).
(c) The length of the sprout that extends the furthest growth caudally measured as the longest straight-line distance caudal to the vagal lobes (FGC). This measure was taken as a *Y*-axis distance (i.e., parallel to the brain-spinal cord axis) from the caudal edge of the vagal lobes to the tip of the most caudally projecting sprout (FGC; Figure 2A, orange sprout). The caudal edge of the vagal lobes marks the rostral boundary of the SML-crush wound.

Other features of M-axon sprouts that were cataloged include reversal of direction, midline crossing, relationship to the first ventral root, formation of a neuroma, and entry into and projection past the wound site.

Two experimenters independently calculated regrowth distances. The more conservative measure between the two was selected. As a result, the measurements are most likely an underestimate of the total regrowth.

### Recording of EMG Responses Evoked by Threshold Stimulation of Mauthner-Axons After Spinomedullary Level-Crush and Return of Startle Responses

Control EMG responses from the left mandibular, trunk and tail musculature evoked by M-axon activation were compared to those elicited after SML-crush. Two fish underwent sham crush operations and were tested for trunk EMG responses 431 days (∼1.2 years) postoperatively to control for the possibility of loss of startle responses with age.

Short-term SML-crush (2 days postoperative) and long-term SML-crush (198–468 days postoperative) fish were anesthetized as described above and the medulla oblongata was exposed between the vagal lobes by removing the “cap” of dental impression material, the wire, and thread that anchored it and by suctioning away the Vaseline-paraffin mixture. Control fish had their brains exposed as described in the “Brain Dissection” section above.

**FIGURE 3:**
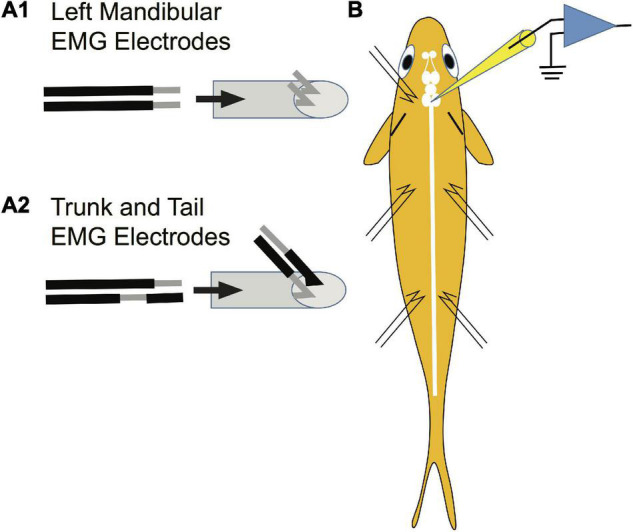
Method used to prepare EMG electrodes. Paired stainless-steel wires were used to record from the mandibular, trunk and tail musculature. **(A1)** Construction of electrodes used in recording EMGs from the left mandibular muscle. Two mm of insulation was scraped off each electrode tip for mandibular electrodes as shown to the left of the arrow. The pair of wires was drawn through a syringe needle as shown to the right of the arrow so that the tips of the wires protruded 2 mm beyond the tip of the syringe and the tips were then bent at a 45° angle relative to the syringe needle and were spread apart. This design risked short circuiting the wires but was required due to the small size of the muscle. **(A2)** The paired electrodes for recording trunk and tail EMGs were constructed differently than those in panel **(A1)** to minimize the possibility of an electrical short circuit between wires (see section “Materials and Methods”). **(B)** Placement of EMG electrodes into the left mandibular muscle and bilaterally into the trunk and tail musculature.

Paired stainless-steel wires (insulated 42-gauge wire) were used to record from the left mandibular and trunk and tail musculature bilaterally. The mandibular EMG electrodes were constructed differently than those for trunk and tail musculature due to the small size of the mandibular muscle. Specifically, 2 mm of insulation was scraped off each electrode tip for mandibular electrodes (Figure 3A1 to the left of the arrow). The pair of wires was drawn through a syringe needle (21 gauge, 25.4 mm) so that the tips of the wires protruded 2 mm beyond the tip of the needle and the tips were then bent at a 45° angle relative to the syringe needle and the tips were spread apart (Figure 3A1 to the right of the arrow). In contrast, the paired electrodes for recording trunk and tail EMGs were constructed to minimize the possibility that the bared portion of the wires would touch. The insulation was scraped off one of the paired wires 2 mm from the tip of the wire while 2 mm of insulation was scraped off the other wire of the pair, but at a distance of 2.5 mm from the tip of the wire (Figure 3A2 to the left of the arrow). The pair of wires was drawn through a syringe needle and the tips of the wires were bent 4.5 mm from the end of the wires so that the tips formed a 45° angle with respect to the long axis of the syringe needle (Figure 3A2 to the right of the arrow). EMG electrodes were placed into musculature while fish were under general anesthesia. EMG electrodes were inserted into the left mandibular musculature and bilaterally into the trunk and tail musculature. Before placement, one or two scales were removed from the sites of insertion. To reduce variability in the experiments, the EMG electrodes were always inserted by one experimenter (SZ). The syringe needle with the mandibular electrodes was inserted at a 45° angle to the main axis of the fish and then carefully withdrawn leaving the tips of the wires “harpooned” into the muscle. Trunk and tail electrode pairs were inserted at a 45° angle to the main axis of the fish in a rostral direction into musculature dorsal to the lateral line. The trunk insertion site was at the rostral edge of the dorsal fin [2.3 ± 0.2 cm (*n* = 23) caudal from the brain recording site] while that of the tail was the caudal edge of the dorsal fin [5.4 ± 0.4 (*n* = 23) cm caudal from the brain recording site]. The placement of EMG electrodes is shown in the schematic of Figure 3B. The wires were secured to the operating chamber with tape. The insulation on the opposite ends of the wires was burnt, the bare wire was burnished with sandpaper, and then connected to an extracellular amplifier.

### Recording From the Mauthner-Axon

After insertion of the EMG electrodes and exposure of the brain, topical anesthetic (20% benzocaine in a water-soluble glycol base; ULTRA-CARE Ultradent Products) was placed over the wound area and care was taken to prevent the local anesthetic from touching the brain. The water with general anesthetic was replaced with anesthetic-free water and the fish regained respiratory movements and were ready for recording in about 15 min. Topical anesthetic was reapplied to the skull wound about every 15–20 min.

The tip of a glass microelectrode (filled with 3 M KCL; 3–7 MΩ) was lowered to the surface of the medulla oblongata just caudal to the facial lobe. The electrode was positioned over one of the M-axons which are visible at this brain level with the aid of a dissecting microscope (see Figure 5 in Zottoli et al., 1994). Other features that helped us ensure that we were recording from the M-axon included:

(a) A depth of about 100 and 150 μm below the brain surface.
(b) An intracellular microelectrode excursion of greater than 40 μm without a significant drop in the RMP.
(c) The ability to re-penetrate the axon repeatedly.
(d) The presence of excitatory post-synaptic potentials in response to clapping.
(e) Movement of the fish on threshold depolarization of the M-axon.

Recordings of EMG responses to threshold depolarization of the M-axon were generally recorded after initial penetration when the resting potential was optimal. Fish movement resulted in a reduction of resting potential which in many, but not all cases, regained initial levels.

After electromyographic experiments, general anesthetic was resumed and some of the axons were filled with Lucifer yellow dye. After injection, the anesthetized fish were perfused through the heart with 100 mL of 10% formalin in phosphate buffer (Fisher Scientific, Inc.). The brains were removed and placed in fresh fixative overnight, dehydrated, and cleared in methyl salicylate for observation of M-axon regrowth in brain wholemounts. The brains were later embedded in paraffin and transverse sections (15 μm) were mounted on glass slides and stained with cresyl violet acetate.

In two free swimming fish, trunk EMG responses were recorded during acoustic/vibratory-evoked startle responses. These recordings were compared to those evoked in the same fish by intracellular M-axon activation.

### Selective Axotomy Technique

A glass microelectrode (5–10 MΩ) filled with 3 M KCl was placed on the brain over the M-axon. Once penetrated, an axon was selectively axotomized by gently tapping the manipulator until the resting potential (initially around −75 mV) was reduced and stabilized below −30 mV for at least 4 min. This technique was used prior to Lucifer yellow injections of the proximal and distal segments of the M-cell (see Figure 9). A more detailed account of this technique can be found in Koganti et al. (2020).

### Double Mauthner Cell Ablation Followed by Spinomedullary Level-Crush

Double M-cell ablation followed by SML-crush (ablation-crush) operations were designed to determine whether post-injury startle responses occur in the absence of M-cells. M-cell somata were located with a microelectrode by stimulating their axons in the spinal cord. The antidromic action potential generates a short-latency, extracellular field potential that can be up to −40 mV in amplitude within the axon cap (a specialized structure surrounding the initial-segment axon hillock region of an M-cell; Furshpan and Furukawa, 1962). This electrophysiological “signature” provides a point of reference from which any part of the soma, the two major dendrites, and axon can be located. A microelectrode (filled with 3 M KCl; 3–7 MΩ) initially penetrated the surface of the medulla oblongata about 400 μm to one side of the midline and at the rostro-caudal level where the corpus cerebellum joins the medulla. Electrode tracks about 1.5 mm in depth were used to search for the antidromically evoked field potential in the M-cell’s axon cap (Furshpan and Furukawa, 1962). The criteria for localizing the axon cap was set as an extracellular field potential of 15 mV or more (Furshpan and Furukawa, 1962). Once this site was localized, the electrode was removed and reinserted into the brain 50 μm laterally. The M-cell soma was identified by its depth (1.5 mm), and its short-latency extracellular field potential that was smaller than that in the axon cap (i.e., less than 5 mV; see Figure 4 of Zottoli et al., 1999). Identification of the M-cell was confirmed after intracellular penetration by the occurrence of both postsynaptic potentials elicited by auditory stimulation (i.e., clapping) and the short-latency action potential evoked by antidromic stimulation. The manipulator was then tapped so that the electrode mechanically disrupted the membrane of the cell. Once the resting potential was less than 30 mV with a concomitant decrease in action potential amplitude and remained low for at least 4 min, the cell was considered ablated. During this interval, the electrode was occasionally lowered through the cell so that the soma was “skewered” and the manipulator was again tapped. Subsequently, the electrode was moved to the other side of the medulla and the other M-cell was located and ablated in the same way. After the double ablation was complete, the spinal cord was crushed at the SML. Fish that had the brain exposed and sealed acted as controls. More detailed information on the ablation technique can be found in Zottoli et al. (1999).

To assess whether ablation resulted in M-cell death, fish were sacrificed under anesthesia (0.024% ethyl-m-aminobenzoate) after the last set of behavioral trials. When respiration had ceased, they were perfused through the heart with 100 ml of 10% formalin in phosphate buffer (Fisher). The brains were removed and placed in fresh fixative overnight, dehydrated, cleared in methyl salicylate, and embedded in paraffin. Transverse sections (15 μm) were mounted on glass slides and stained with cresyl violet acetate.

### Dextran Biotin Backfilling of the Mauthner Cell

Dextran biotin was dissolved and recrystallized on the tip of a 45-gauge stainless steel wire. Under general anesthesia, the brain of an adult goldfish was exposed (see above), the rostral spinal cord was transected, and the dextran biotin was placed on the rostral stump of the cord until it dissolved. The brain was sealed and the water circulating over the gills was replaced with anesthetic-free water. Once the fish recovered, it was placed in its home tank. Two days postoperatively the fish was re-anesthetized and perfused through the heart with 4% paraformaldehyde, 1% glutaraldehyde in 0.1 M phosphate buffer, pH 7.4. The brain was removed and processed as described in Gilland et al. (2014).

### Statistics

Unpaired *t*-tests were performed on variables with normal distribution with equal variance and unpaired *t*-tests with Welsh’s correction were performed for those groups with unequal variance. For groups with non-normal distributions a Mann-Whitney test was performed. Normality tests were performed using the Shapiro-Wilk test. The significance level for all tests was set at *P* = 0.05. The statistical program used was GraphPad Prism version 9.0.2 for Windows, GraphPad Software, San Diego, CA, United States, www.graphpad.com.

## Results

### Regrowth of Mauthner-Axons 56–85 Days After Spinomedullary Level-Crush

Regrowth of M-axons was studied after whole spinal cord crush at the junction of the spinal cord and medulla oblongata (spinomedullary level, SML; see Figure 1). A total of 22 M-axons in 15 fish were filled with Lucifer yellow between 56 and 85 days postoperatively, an interval when post-injury startle responses can be triggered by an abrupt acoustic/vibratory stimulus (see Figure 5 of Zottoli and Freemer, 2003). The methods used to measure sprouts are shown in Figure 2A and the mean values of sprout lengths in Figure 2B represent the GGC (range = 476–4711 μm), GGR (range = 273–4149 μm), and the furthest growth caudally measured as the longest straight-line distance caudally from the rostral edge of the SML-crush wound (FGC; range = 367–3034 μm).

**FIGURE 4:**
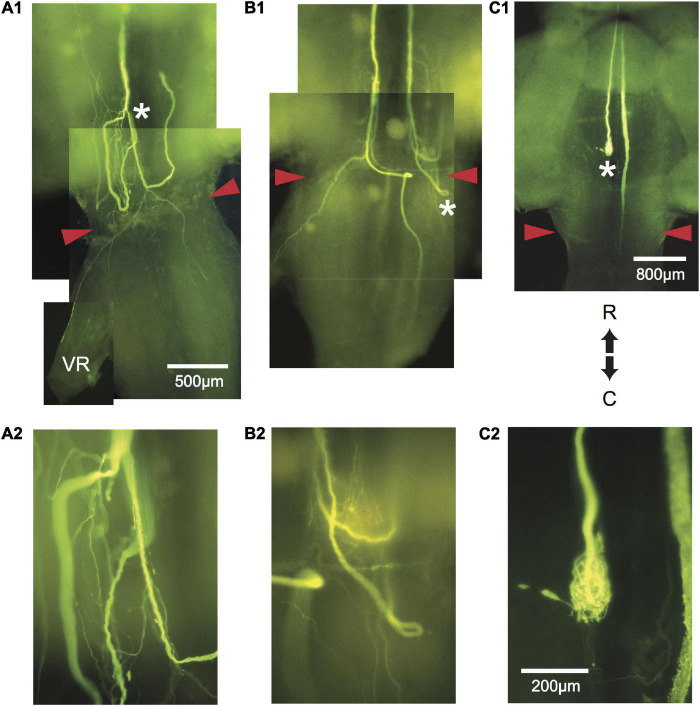
Aberrant pathway choice of axotomized M-axons. **(A1)** Sprouting of the left M-axon of a fish 66 days postoperatively. The photographic montage of brain wholemounts shows reversal of direction (from caudal to rostral) of a number of sprouts anterior to the level of the wound site (marked by red arrowheads in this and subsequent panels), a midline crossing from left to right, a sprout that projects out the left ventral root (VR), and sprouts that project caudally past the wound site. The area to the left of the asterisk of panel **(A1)** is enlarged in panel **(A2)**. **(B1)** Montage illustrating regrowth of both M-axons in a fish 63 days postoperatively. The right axon has at least two sprouts that reverse direction (caudal to rostral) rostral to the wound and one that reverses within the wound. The left axon crosses the midline within the wound and projects caudally and laterally. A sprout on the left side projects out the ventral root. The area above the asterisk in panel **(B1)** is enlarged in panel **(B2)**. **(C1)** The left M-axon of a fish 78 days postoperatively appears to have no regrowth. However, in the higher magnification of panel **(C2)**, it is clear that the axon has formed a neuroma. Only the sheath of the right axon has been filled with dye. R, rostral; C, caudal. All photographs are from brain wholemounts rostrally toward the top and caudally toward the bottom.

Regrowth of one axon formed a neuroma. Of the remaining 21 axons, regrowth was initiated from the retracted tip as “parent” branches (range = 1–7; 3.1 ± 2, mean ± SD). Many sprouts that emanated from these branches chose aberrant pathways as shown in Table 1. Sprouts project rostrally, start in one direction and then reverse their trajectory, abut or enter the ventral root, cross the midline, or form a neuroma. In general, sprouts reverse direction from a caudal trajectory to a rostral one just rostral to the wound and cross the midline in the vicinity of the wound. Multiple sprouts from the same axon can follow aberrant paths. For example, 15 axons that had at least one rostrally projecting sprout also had one that projected caudally and 3 of these axons also had sprouts that abutted or entered the ventral root. Overall, 82% of axons had at least one sprout that entered the wound. We estimate that the wound extends for less than 1 mm and that at least 36.4% of axotomized M-axons have at least one sprout that projects caudally to the wound site. In brain wholemounts, the sprouts that traverse the wound appear lateral to the normal M-axon trajectory. This lateral position was confirmed in cross-sections in five fish (see Figures 8D2, 9C4). The aberrant pathway choice of M-axon sprouts for three fish is shown in Figure 4. All images are from brain wholemounts. Regrowth of the left M-axon of a fish 66 days postoperatively, as shown in the photographic montage of Figure 4A1, illustrates the reversal and rostral projection of a number of sprouts anterior to the level of the wound site (designated by red arrowheads), a sprout that crosses the midline from the right side of the spinal cord to the left and then projects out the left ventral root, and sprouts that project caudally past the wound site. The area to the left of the asterisk in Figure 4A1 is enlarged in 4A2 to show the extent of sprouting. Both M-axons in a fish 63 days postoperatively are shown in Figure 4B1. The right axon has sprouts that reverse direction rostral to and within the wound; the sprouts then project rostrally. A sprout from the left axon bifurcates and one branch crosses the midline within the wound and projects caudally and laterally on the right side of the spinal cord while the other branch projects out the left ventral root. The area above the asterisk in Figure 4B1 is enlarged in 4B2 to highlight the extent of sprouting in that region. The left M-axon of a fish 78 days postoperatively appears to have no growth in Figure 4C1 (above the asterisk). However, it is clear that when the micrograph is enlarged (Figure 4C2), the axon has sprouts that form a neuroma. Only the sheath of the right M-axon was filled with dye.

**TABLE 1:** Projection patterns of Mauthner axon (M-axon) sprouts 56–85 days following spinomedullary level (SML)-crush.

Percentage of 22 axons filled with Lucifer yellow that had at least one sprout that:					
Projects rostrally	Projects caudally past the vagal lobes*	Projects caudally past the wound site**	Reverses direction	Abuts or enters the first ventral root	Projects across the midline
68.2%	81.8%	36.4%	59.1%	31.8%	31.8%

*This brain level marks the rostral edge of the wound site.

**This calculation is based on our estimation that the wound is about 1 mm in length.

### The Emergence of Post-injury Startle Responses

Experimental fish were monitored for post-injury startle responses to a tap on their tank. Once a response was elicited, fish were tested with the acoustic/vibratory stimulus (see section “Materials and Methods”) in blocks of six trials with a 2 min inter-trial interval. Experimental fish were tested between 198 and 213 days postoperatively and the responses were compared to those of sham-operated control fish tested between 329 and 421 days postoperatively (Table 2). Experimental fish had a significantly lower frequency of response (*P* = 0.02) and latency from stimulus onset to response (*P* = 0.03), a smaller escape trajectory angle (*P* = 0.03), and straight-line center of mass movement (*P* = 0.046) as compared to sham-operated controls. The linear velocity of the center of mass was not significantly different between the two groups (*P* = 0.1).

**TABLE 2:** Comparison of startle response parameters between SML-crush and sham-operated control fish.

Fish	*n*	Postoperative interval (days)	Responsiveness (%)	Latency (ms)	ETA (°)	Straight line (cm)	Velocity (cm/s)
SML-crush	4	198–213	12.54.9	44.113.7	58.337.2	2.41.2	46.19.5
Control*	8	329–421	70.817.2	18.42.7	101.720.5	3.50.6	63.715.1

*These fish were the same sham-operated control fish used in Zottoli and Freemer (2003) but at longer postoperative intervals.

### Control and Experimental EMG Responses Evoked by Intracellular Mauthner-Axon Stimulation

Intracellular stimulation of an M-axon in control fish activates cranial muscles of the jaw, eyes, opercula, and pectoral fins bilaterally (supraspinal head component; Auerbach and Bennett, 1969; Diamond, 1971; Hackett and Faber, 1983) as well as ipsilateral trunk and tail musculature. The placement of EMG electrodes in the left mandibular muscle to monitor the supraspinal head component and in trunk and tail musculature on each side of a fish is shown in Figure 3B.

Threshold stimulation of control axons resulted in visible movement of head, trunk, and tail in fish treated with topical anesthetic. Activation of 28 M-axons in 15 fish elicited trunk and tail EMG responses. Eight of these control axons did not elicit left mandibular EMG responses. Care was taken to stimulate intermittently to reduce the possibility of synaptic fatigue. We believe that the occasional failure to record left mandibular EMGs in control and experimental fish resulted from “shorting” of the recording wires because, (1) visible head level movement occurred during M-axon stimulation despite no left mandibular muscle recording, (2) in some cases the left mandibular EMG was initially present and then lost on subsequent stimulation (compare A1 with C1 and A2 with C2 in Figure 8) and, (3) in control fish, trunk and tail responses were present in all cases when the left mandibular responses were absent (see methods for a description of EMG electrode construction).

**FIGURE 5:**
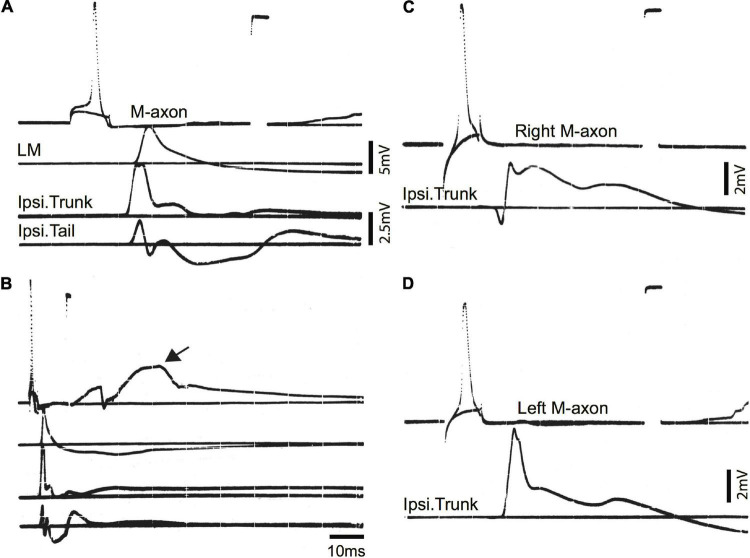
Mauthner-axon activation and EMG responses in control fish. **(A)** Intracellular activation of the M-axon results in EMG responses of head, trunk, and tail musculature. Traces from top to bottom: (1) Intracellular recording from the M-axon above and below threshold. Unless stated otherwise, calibration pulse is 80 mV and 1 ms in this and subsequent figures, (2) EMG recording from the left mandibular muscle (LM), (3) recording of trunk musculature ipsilateral to the M-axon (Ipsi. Trunk), and (4) EMG recording from the tail musculature ipsilateral to the M-axon (Ipsi. Tail). **(B)** Recording, as in panel **(A)**, but at a longer time base to show the time course of EMG responses. The arrow designates the shift in resting potential due to movement of the fish. **(C,D)** Activation of the right **(C)** and left **(D)** M-axons in a control fish, held for 422 days in captivity. The top trace is from the M-axon above and below threshold and the bottom trace is an EMG recording from trunk musculature ipsilateral to the M-axon.

Examples of control EMG recordings are presented in Figure 5. Recordings shown in A and B are the same but B has a longer time base to demonstrate the duration of EMG responses. The movement of the fish oftentimes resulted in a depolarizing shift in the resting membrane potential (arrow in B). The time from spike initiation until the first signs of this shift was 9.3 ± 3.2 ms (mean ± S.E.; *n* = 18). Subthreshold and threshold traces are superimposed, and include from top to bottom: Intracellular recording from the M-axon, left mandibular EMG, trunk musculature EMG ipsilateral to the M-axon, and tail musculature EMG ipsilateral to the M-axon. Two sham-operated fish were tested 422 days (∼1.2 years) postoperatively to ensure that the control EMG responses do not deteriorate with age, or that there might be effects that result from the operation. Activation of the right (C) and left (D) M-axons (upper traces) resulted in ipsilateral trunk EMG responses (lower traces). Head, trunk, and tail movement was visible when M-axons were brought to threshold and the EMG responses were within the range of amplitudes recorded for control fish that did not have a sham operation.

**FIGURE 6:**
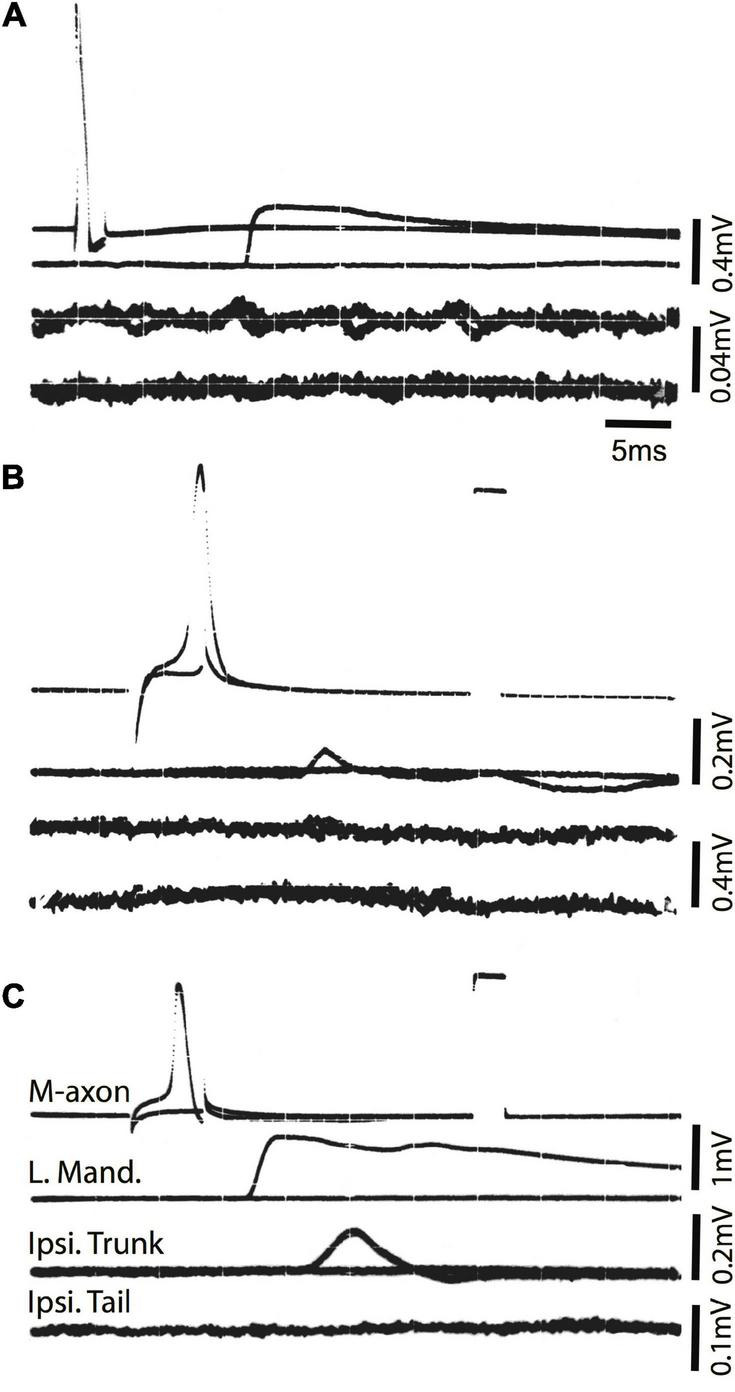
Activation of the M-axon after spinal cord crush and the emergence of post-injury startle responses results in little or no EMG responses in trunk and no response in tail musculature. **(A)** Recordings made from a fish 2 days after SML crush to determine whether the injury severed the M-axons. Traces from top to bottom: (1) Intracellular recording from the M-axon above and below threshold, (2) EMG recording from the left mandibular muscle, (3) EMG recording from the trunk musculature ipsilateral to the M-axon and (4) EMG recording from the tail musculature ipsilateral to the M-axon. The activation of an M-axon resulted in left mandibular EMGs but no response in the ipsilateral trunk or tail musculature. **(B)** An M-axon action potential resulted in a left mandibular EMG but no trunk or tail EMGs 206 days postoperatively. **(C)** An M-axon action potential resulted in a left mandibular EMG and a small EMG in the trunk but not tail musculature 213 days postoperatively.

To ensure that the SML-crush wound was effective in separating M-axons, nine axons in seven fish were studied electrophysiologically 2 days after SML-crush. Stimulation of an M-axon to threshold resulted in visible movement confined to the head. Three of the nine axons elicited very small trunk EMG responses (0.11, 0.014, and 0.014 mV) and none of the axons elicited EMG responses in the tail musculature. An example of a recording from a SML-crush fish after 2 days is shown in Figure 6A. The activation of an M-axon (top trace) resulted in a left mandibular EMG but no response in the ipsilateral trunk or tail musculature.

**FIGURE 7:**
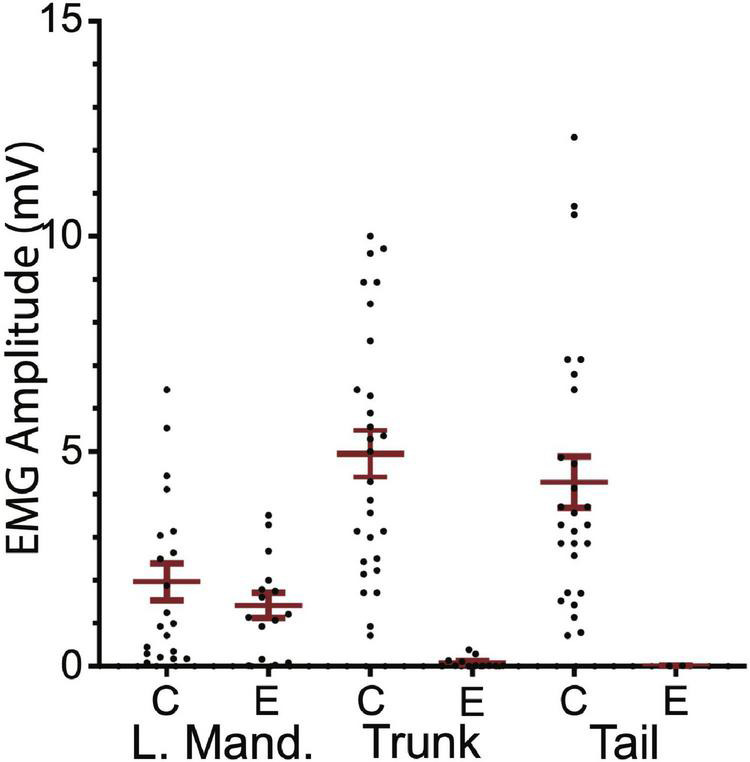
Comparison of control and experimental EMG responses elicited by M-axon activation. Comparisons of control (C) and experimental (E) EMG responses of left mandibular muscle (L.Mand.) and trunk and tail musculature. Left mandibular control and experimental EMG amplitudes (mean ± S.E.M.) were not significantly different (*P* = 0.50) while the control trunk and tail EMGs were significantly greater (*P* < 0.001) than those of experimental fish.

Ten SML-crush fish displayed post-injury startle responses and were tested for EMG responses evoked by M-axon activation 198–468 days (∼0.5–1.3 years) postoperatively. Activation of an M-axon resulted in a visible head component movement. Eighteen axons in ten fish were studied and fifteen of the axons evoked mandibular EMG responses. The SML-crush trunk EMGs evoked in 18 axons can be lumped into three categories: (1) no detectable response (6 axons), (2) detectable EMG ≤ 0.019 mV (8 axons), and (3) peak EMG between 0.107 and 0.386 mV (4 axons); by comparison the smallest control trunk EMG response was 0.7 mV. In two M-axons, EMG responses were recorded in the tail musculature (0.014 and 0.011 mV). Examples of recordings from SML-crush fish where no EMG responses were recorded are presented in Figures 6B,C. Activation of the M-axon resulted in a mandibular muscle EMG, but no response in ipsilateral trunk or tail musculature for a fish 206 days postoperatively (B). In contrast, one of the larger trunk EMG recordings from a fish 213 days postoperatively is shown in Figure 6C. The mean amplitude of left mandibular, trunk, and tail EMG responses for control and SML-crush fish are compared in Figure 7. While there was no significant difference between the left mandibular EMG amplitudes between control and SML-crush animals (*P* = 0.50), stimulating the axons of control fish elicited significantly larger trunk and tail EMG responses than those of experimental fish (*P* < 0.001). The SML-crush left mandibular and trunk EMG latencies were significantly longer when compared to controls (*P* = 0.03 and 0.01, respectively).

**FIGURE 8:**
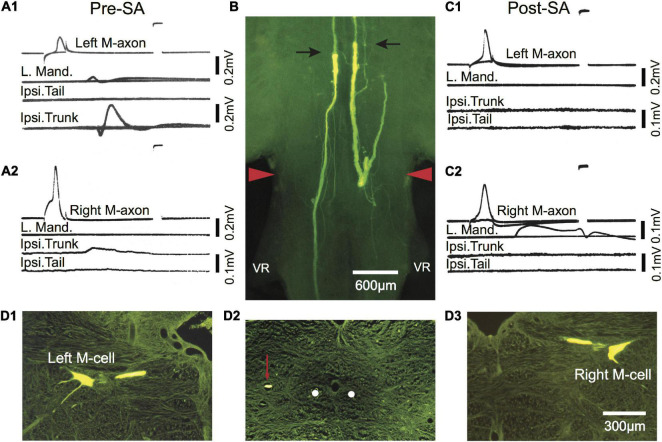
Post-injury trunk EMG responses evoked by threshold stimulation of the M-axon are abolished after selective axotomy. **(A1,A2)** EMG recordings evoked by stimulation of the left **(A1)** and right **(A2)** M-axons 433 days postoperatively resulted in EMG responses in the trunk but not tail musculature. Note that the tail EMG is above the trunk EMG in panel **(A1)**. **(B)** Lucifer yellow-filled distal segments of selectively axotomized M-axons in a brain wholemount. The axons were axotomized after the recordings were made in panels **(A1,A2)** (Pre-SA). The top of the photograph shows the tips of the distal segments marked with arrows with only the sheath visible rostrally (i.e., above the arrows; proximal segment is not visible). The wound site is delineated by red arrowheads. **(C1,C2)** Stimulation of the proximal portion of the M-axon 13 days after selective axotomy (Post-SA). An M-axon spike in either axon did not elicit EMG responses in the trunk as it had pre-selective axotomy. **(D1–D3)** Cross sections (15 μm) of the brain shown in the wholemount of panel **(B)**. **(D1,D3)** Left and right Mauthner cells filled by iontophoresis of Lucifer yellow into the proximal segments of the M-axons after selective axotomy. **(D2)** A cross section of the spinal cord just caudal to the first ventral root (VR), showing the position of the left axon sprout (red arrow) lateral and dorsal to the position the M-axons follow in control fish (white dots).

**FIGURE 9:**
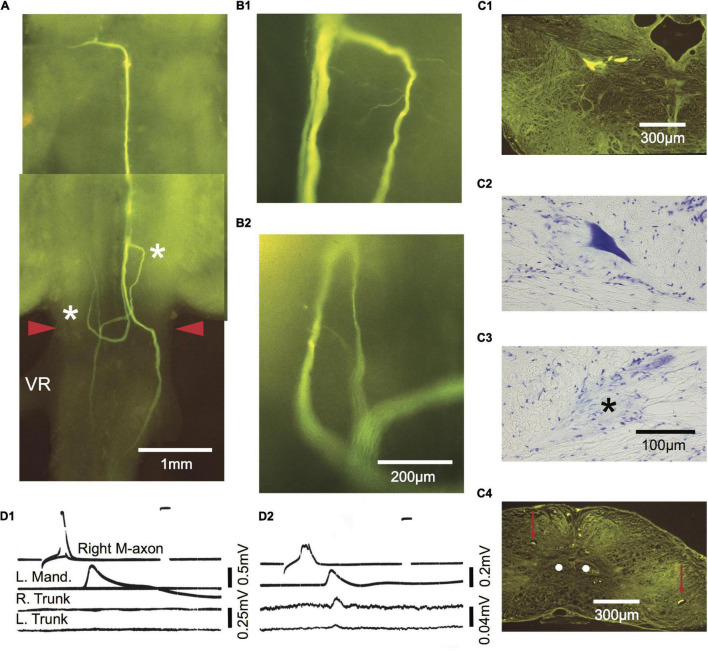
Comparison of M-axon evoked EMG responses with regrowth patterns 434 days after a SML crush. Photographic montage of a Lucifer yellow-filled M-cell as seen in a brain wholemount. Three parent sprouts emanate from the M-axon. One remains ipsilateral and projects caudally. A second crosses the midline in the wound and projects both rostrally and caudally. A third crosses the midline, projects caudally, and then reverses direction rostrally at the anterior margin of the wound. The area to the left of the asterisk on the right side in panel **(A)** is enlarged in panel **(B1)**. The area to the right of the asterisk on the left side in panel **(A)** is enlarged in panel **(B2)**. A fine sprout appears to have a growth cone at its tip. **(C1–C4)** Cross sections of the wholemount brain shown in panel **(A)**. Left M-cell body filled with Lucifer yellow **(C1)** and stained with cresyl violet **(C2)**. **(C3)** An axon cap (*) was found at the former location of the right cell body. **(C4)** Cross section caudal to the wound site showing a sprout on the left and right of the spinal cord (red arrows) that corresponds to those seen in the wholemount. The sprouts are not located near the normal projection pathway of the M-axons (white dots). **(D1,D2)** Activation of the M-axon and EMG recordings from left mandibular and right and left trunk musculature. The recording in panel **(D1)** did not elicit a trunk response while somewhat later **(D2)** after the action potential had deteriorated, small EMGs were recorded in both the right and left trunk musculature.

In one fish activation of the left and right M-axons 433 days (∼1.2 years) after SML crush resulted in ipsilateral trunk EMGs as shown in Figures 8A1,A2 (Pre-SA). To determine whether the M-axons were responsible for the trunk EMGs, both axons were selectively axotomized and 13 days post-axotomy (Post-SA) activation of the proximal M-axon segments did not elicit ipsilateral trunk or tail EMGs (Figures 8C1,C2). The success of the selective axotomy procedure is apparent after Lucifer yellow fills of the distal segments of M-axon in Figure 8B. There was a correlation of the amplitude of the EMG and the length of regrowth caudally; that is, the left M-axon projected further caudally and had a larger trunk EMG compared to that of the right M-axon. Iontophoresis of Lucifer yellow into the proximal M-axon segments resulted in the filling of the left (D1) and right (D3) M-cells as shown in cross sections. The left sprout is shown caudal to the wound in the cross section of Figure 8D2. The sprout (red arrow) is lateral to the normal M-axon pathway (shown as white dots).

A comparison of M-axon regrowth patterns and EMG responses 434 days postoperatively is presented in Figure 9. The photographic montage of the right axon in Figure 9A shows three parent sprouts. One remains ipsilateral and projects caudally. A second crosses the midline in the wound and projects both rostrally and caudally. A third crosses the midline, projects caudally, and then reverses direction rostrally at the anterior margin of the wound. The area to the left of the more rostral asterisk (on the right) is enlarged in B1 and the area to the right of the more caudal asterisk (on the left) is enlarged in B2. Fine sprouts are visible, some of which appear to have a growth cone on the tip. Cross sections of the left cell body filled with Lucifer yellow and stained with cresyl violet are presented in C1 and C2, respectively. The right cell body could not be found but an axon cap (asterisk) marked the former position of the cell (C3). Left and right sprouts are shown in the cross section (red arrows) in relation to the normal position of the M-axons (C4, white dots). Activation of the right M-axon in D1 resulted in a left mandibular EMG but no response in trunk or tail musculature. Somewhat later as the spike deteriorated, stimulation of the right M-axon resulted in left mandibular and small trunk and tail EMGs (**D2**).

### Comparison of Trunk EMGs Evoked in Free-Swimming Fish During a Post-injury Startle Response Compared to EMGs Evoked by Threshold Stimulation of Mauthner-Axons in the Same Fish

**FIGURE 10:**
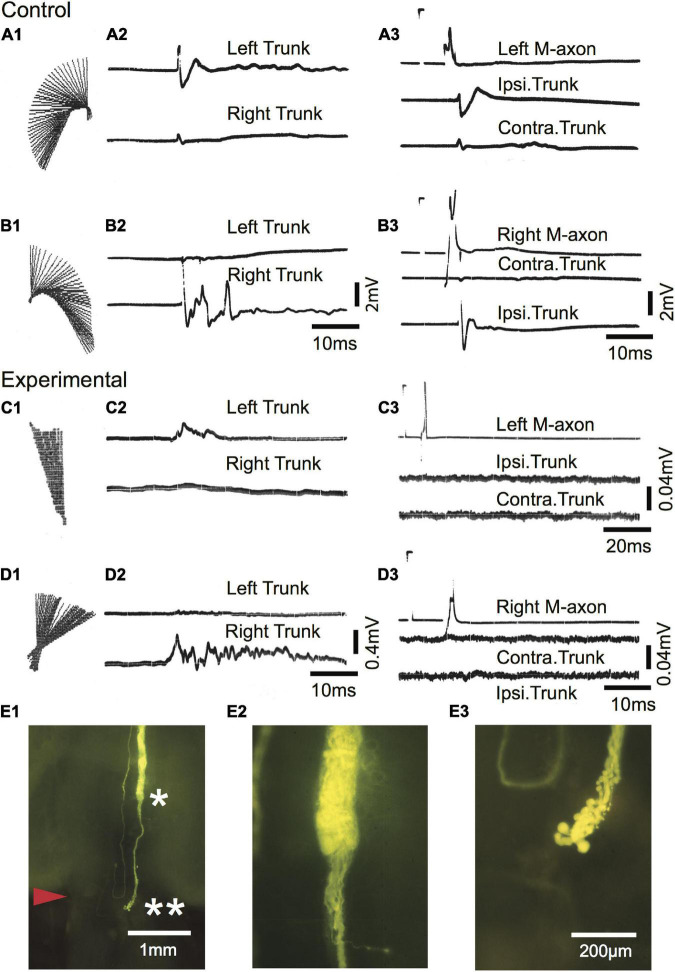
Mauthner-axons do not contribute to the post-injury startle response of a free-swimming goldfish 612 days after SML crush. **(A1–B3)** Sham-operated control fish 588 days postoperatively. **(A1,B1)** C-type startle response to the left **(A1)** and right **(B1)** side in a free-swimming goldfish. Regression lines of the rostral 40% of the fish body are plotted in 2 ms increments [see Figure 2 of Zottoli and Freemer (2003) for a more detailed description]. **(A2,B2)** EMG recordings from left (upper trace) and right (lower trace) trunk musculature during the free-swimming responses shown in panels **(A1,B1)**. The EMGs correspond to the direction of the behavioral response. **(A3,B3)** Fish were moved from the sound test chamber to a holding chamber, and threshold activation of M-axons resulted in ipsilateral EMG recordings. Calibrations in panel **(B2)** are the same for panel **(A2)** and calibrations for panel **(B3)** are the same for panel **(A3)**. **(C1–D3)** Experimental fish 612 days after SML crush. **(C1,D1)** Post-injury startle responses to the left **(A1)** and right **(B1)** side in a free-swimming goldfish. **(C2,D2)** EMG recordings from left (upper trace) and right (lower trace) trunk musculature during the free-swimming responses shown in panels **(C1,D1)**. The EMGs correspond to the direction of the behavioral response. **(C3,D3)** Fish were moved from the sound test chamber to a holding chamber, and threshold activation of M-axons did not result in trunk EMGs. Calibrations in panel **(D2)** are the same for panel **(C2)**. The calibration pulse on the axon trace of C3 is 60 mV, 1 ms and 40 mV, 1 ms for panel **(D3)**. **(E1–E3)** Lucifer yellow fill of the right axon of the experimental fish. **(E1)** Low power image of the brain wholemount. An enlargement of the region to the left of the single asterisk is shown in panel **(E2)** and to the left of the double asterisk in panel **(E3)**.

Startle responses evoked by an abrupt vibratory/acoustic stimulus while the fish were free-swimming are shown in Figures 10A1–D1 as regression lines of the rostral 40% of fish midlines plotted every 2 ms for a sham-operated control [Figures 10A1,B1; 588 days (∼1.6 years) postoperatively] and SML-crush fish (C1,D1; 612 days postoperatively). The corresponding EMGs for left (upper trace) and right trunk musculature (lower trace) are shown in A2–D2. Fish were then moved to a holding chamber, the brain was exposed, and M-axons were penetrated with a microelectrode and brought to threshold. Control EMG responses to left (A3) and right (B3) M-axon stimulation result in ipsilateral trunk responses. Experimental responses to left (C3) and right (D3) M-axon stimulation did not elicit trunk EMGs. The right axon of the experimental fish was successfully filled with Lucifer yellow and is shown in the brain wholemount (Figure 10E1). The area to the left of the single asterisk is enlarged in E2; axonal sprouts have formed a neuroma and some of those sprouts project caudally (double asterisk of E1) which is enlarged in E3.

### Morphology of Mauthner Cells After Spinomedullary Level-Crush Wounds

**FIGURE 11:**
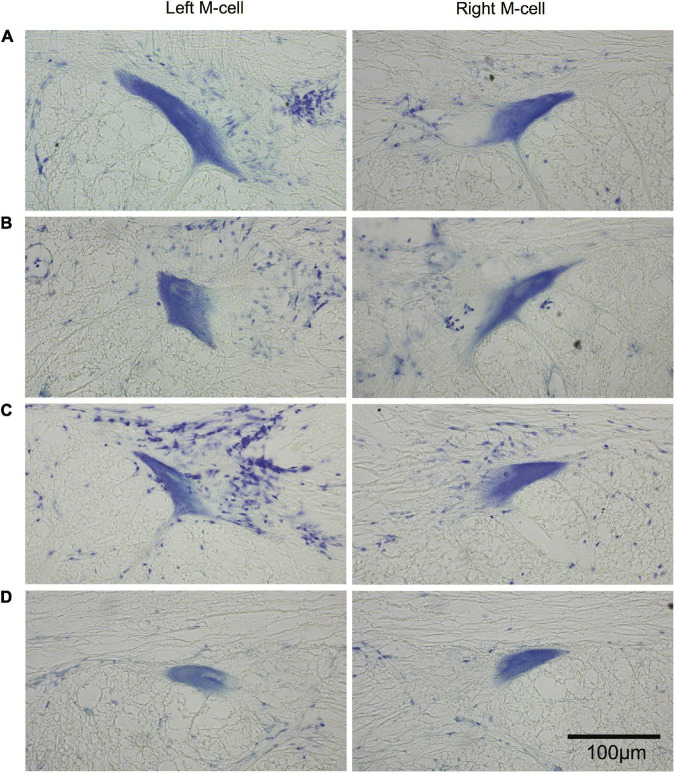
Long-term survival of the Mauthner cell after spinal cord crush. Cross sections (15 μm) of left and right M-cell somata for fish that have undergone SML crush with post-injury startle responses. **(A–D)** Left and right M-cells are presented for four experimental fish 198–214 days postoperatively that were used in the EMG studies. Sections are stained with cresyl violet.

The morphology of M-cell somata was assessed in cross sections (15 μm) of paraffin-embedded brains stained with cresyl violet. Left and right M-cell somata are shown in Figure 11 for four fish (A–D) that had undergone SML-crush, displayed post-injury startle responses, and were used in EMG studies (198–214 days postoperatively). None of the M-cells were swollen or appeared chromatolyzed.

### The Occurrence of Post-injury Startle Responses After Ablation-Crush

Mauthner cells can be located with a microelectrode by the large extracellular field potential evoked by antidromic activation of their axons (Furshpan and Furukawa, 1962). After intracellular penetration, the soma can be mechanically ablated (Zottoli et al., 1999). Four fish had double M-cell ablations followed by an SML-crush (ablation-crush fish). These fish first regained equilibrium and somewhat later displayed startle responses, 61–253 days postoperatively. Frequency of response (responsiveness) and kinematic response parameters are compared between four ablation-crush fish tested 291–437 days postoperatively and four fish tested 198–213 days after SML-crush in Table 3.

**TABLE 3:** Comparison of startle response parameters between ablation-crush and SML-crush fish.

Fish category	*n*	Postoperative interval (days)	Responsiveness (%)	Latency (ms)	ETA (°)	Straight-line (cm)	Velocity (cm/s)
Ablation-crush	4	291–437	18.319.9	401.6	36.77.3	1.80.5	29.18.5
SML-crush	4	198–213	12.54.9	44.113.7	58.337.2	2.41.2	46.19.5
Controls*	8	329–421	70.817.2	18.42.7	101.720.5	3.50.6	63.715.1

*These fish were the same sham-operated control fish used in Zottoli and Freemer (2003) but at longer postoperative intervals.

The frequency of response of ablation-crush fish (*P* = 0.89), latency from stimulus onset to response (*P* > 0.99), escape trajectory angle (*P* = 0.33), straight-line center of mass movement (*P* = 0.45), and linear velocity of the center of mass (*P* = 0.053) are not significantly different from SML-crush fish.

**FIGURE 12:**
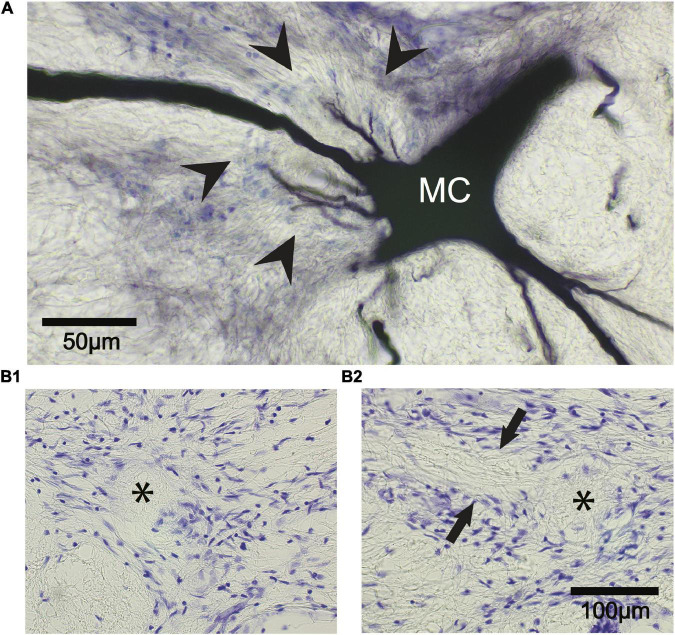
The Mauthner cell (M-cell) axon cap is recognizable 742 days after M-cell ablation. **(A)** A control M-cell filled with dextran biotin highlights the location of the axon cap. The initial segment of the M-axon projects from the cell body (MC) to the left through the center of the cap that is delineated by glial cell nuclei stained with cresyl violet (arrowheads). Cap dendrites project from the axon hillock into the outer portions of the cap. **(B1,B2)** The ablation of both M-cells results in the death and disappearance of the M-cell but the axon cap remains. **(B1)** Left M-cell cap (asterisk). **(B2)** Right M-cell cap (asterisk). Fibers (between arrows) appear to enter the cap from the left side of the photograph. These fibers are presumed to be axons of spiral fiber neurons.

The fish were placed under general anesthesia 740–783 days (∼2–2.2 years) postoperatively and perfused through the heart with 10% formalin in phosphate buffer (pH = 7.4). Brains were embedded in paraffin, sectioned, and stained with cresyl violet to confirm that the M-cells were missing. In all cases, M-cells could not be found while the axon cap was located in 7 out of 8 cells that were ablated. The axon cap, as delineated by arrowheads in Figure 12A, surrounds the initial segment of the M-axon. Cap dendrites extend from the axon hillock into the peripheral portion of this structure. The left (asterisk in B1) and right (asterisk in B2) axon cap marks the former location of M-cells in one fish. Fibers that appear to enter the cap can be seen between arrows. These fibers are presumed to be axons of spiral fiber neurons.

## Discussion

The identifiability of Mauthner cells and their role in the initiation of fast C-starts make this a useful preparation for the study of regeneration in the central nervous system of vertebrates. After spinal cord injury of the adult goldfish at the spinomedullary level (SML-crush), there is an emergence of post-injury startle responses but they are not as frequent, fast, or robust when compared to sham-operated control fish (Zottoli and Freemer, 2003). We have utilized morphological, behavioral, and electrophysiological approaches to show that although the M-cell survives over long postoperative intervals, maintains supraspinal synaptic connections, and maintains extensive regrowth, its activation at most elicits occasional, small EMG responses in trunk and tail musculature.

### Most Mauthner-Axon Sprouts Choose Aberrant Pathways

Mauthner-axon sprouts between 56 and 85 days postoperatively deviate from the normal, caudal trajectory within the fasciculus longitudinalis medialis. Sprouting of the M-axon occurs days after SML-crush at 22°C in the adult goldfish (Koganti et al., 2020). Sprouts branch, cross the midline, project rostrally, abut and enter the first ventral root, form neuromas, and choose caudal pathways lateral to the normal M-axon trajectory. These aberrant choices are maintained for at least 434 days postoperatively and suggest that there is not a preferred pathway. Regenerating fibers project into lateral but not ventral funiculi 6–12 weeks after spinal cord transection in goldfish (Bunt and Fill-Moebs, 1984). The lateral pathway choice of caudally projecting M-axon sprouts in this study corresponds to the findings that M-axon regrowth caudal to a wound in adult zebrafish is not present in the white matter of the ventral spinal cord (Becker and Becker, 2001).

Our observations of branching and aberrant pathway choice of axotomized M-axons are similar to those reported for M-axons in urodele larvae (Holtzer, 1952; Piatt, 1955), larval zebrafish prior to cAMP treatment (Bhatt et al., 2004; Lau et al., 2013), adult goldfish (Al-Goshae and Bunt, 1992; Bentley and Zottoli, 1993; Zottoli et al., 1994; Zottoli and Faber, 2000), and for the M-axon and other large reticulospinal neurons in larval lamprey (Rovainen, 1976; Wood and Cohen, 1979, 1981; Yin and Selzer, 1983; Lurie and Selzer, 1991; Oliphint et al., 2010). The reversal in direction of growth from caudal to rostral and midline crossings are more common proximal or within the wound in adult goldfish, as has also been noted in amphibians (Holtzer, 1952; Lee, 1982) and larval lamprey (Rovainen, 1976; Wood and Cohen, 1979; Yin and Selzer, 1983). Db-cAMP treatment has been shown to eliminate branching or rostral turning in favor of more direct pathways in larval lamprey (Lau et al., 2013) and larval zebrafish (Bhatt et al., 2004).

In an earlier study, 85.6% of sprouts that extended across an SML-crush wound 30–42 days postoperatively were within or in close proximity to the first ventral root (Zottoli et al., 1994). Thirty-two percent of axons had a sprout found in association with the ventral root in this study 56–85 days postoperatively. Since the wound (i.e., SML-crush) and temperature (22°C) were the same in both studies, we speculate that some sprouts that orient toward the ventral root at short postoperative intervals ultimately project elsewhere or sprouts in the ventral root may retract and then choose a different pathway.

### Mauthner-Axon Sprouts Are Capable of Regrowing Across Spinomedullary Level-Crush Wounds

Eighty-two percent of M-axons have a sprout that enters the lesion and 36.4% of M-axons have a sprout that extends caudally to the crush wound site 56–85 days postoperatively. Our measurements are conservative and underestimate the actual directed growth since sprouts labeled with Lucifer yellow were eventually lost in the autofluorescence of the spinal cord. In addition, we chose the more conservative measures of sprout length by two separate experimenters. Nonetheless, sprouts are able to enter the wound site and some extend at least 3 mm caudal to the rostral edge of the wound. This sprouting argues against the inability of M-axons to regrow due to “excessive morphological specialization” (Kiernan, 1979).

The M-axon regrows past spinal cord lesions and forms functional synapses in *Xenopus laevis* tadpoles (Lee, 1982), while the ability of M-axons to regrow in urodele larvae decreases with age (Holtzer, 1952; Piatt, 1955). Larval zebrafish M-axons have the capacity to regrow caudal to a wound and form synapses after laser axotomy (Hu et al., 2018), and such regrowth results in the return of fast C-starts after spinal cord lesions (Bhatt et al., 2004; Hecker et al., 2020a). In contrast, the regrowth in larval lamprey, adult urodeles, and teleost fish is more limited. Reports of axotomized M-cells that lack sprouts have utilized silver-stained preparations which are difficult to interpret since this method limits the ability to detect fine processes (*Xenopus* larvae, Sims, 1962; adult urodeles, Piatt, 1955; adult goldfish, Bernstein, 1964). However, the M-axon was shown to traverse a wound site in one case in an adult urodele (Piatt, 1955). Retrograde labeling has not shown M-axon regrowth to an application site caudal to the wound in some cases (adult goldfish, Sharma et al., 1993; adult zebrafish, Becker et al., 1997) but has in others (Becker et al., 1998; Becker and Becker, 2001).

### Axotomized Mauthner-Cells Maintain Supraspinal Synaptic Connections

Mauthner-cells synapse on cranial relay neurons (CRN) in the brain, and these connections mediate the supraspinal head component of a startle response (Hackett and Faber, 1983). A single M-axon in the hatchetfish bilaterally activates a CRN that synapses on motoneurons in trigeminal, rostral facial, and in some cases oculomotor and trochlear motor nuclei (Auerbach and Bennett, 1969; Barry and Bennett, 1990). In goldfish, activation of an M-axon results in bilateral adduction of jaw, opercula, and eye muscles (Diamond, 1971; Hackett and Faber, 1983). SML-fish maintain a visible supraspinal head component to M-axon activation at all postoperative intervals. Thus, synaptic connections rostral to the wound were maintained over the course of our electrophysiological studies. In addition, seven axons that were tested displayed PSPs in response to clapping between 198 and 214 days postoperatively; such a response implies that synapses between the VIII^th^ nerve and the M-cell are intact.

### Do Some Axotomized Mauthner-Axons Reconnect to Targets That Elicit EMG Responses?

An SML-crush wound consistently results in the severance of the M-axon as demonstrated in many studies by Lucifer yellow fills (Zottoli et al., 1987; Zottoli and Freemer, 2003; Koganti et al., 2020). These axons can regrow across SML-crush sites but it is not clear whether they re-form synapses with targets capable of eliciting EMG responses either rostral or caudal to the wound. Small EMG responses (<0.014 mV) existed 2 days after SML-crush in the trunk but not the tail musculature in 33.3% of the axons studied. Similar small EMG responses were recorded after M-axon stimulation in trunk (8 of 18 axons; 198–468 days postoperatively; range = 0.006–0.019 mV) and tail musculature (2 of 16 axons; 0.007 and 0.014 mV). We speculate that these very small EMGs are a result of volume conduction from EMGs in the head region, although we cannot eliminate the possibility that non-M-cell axons were spared during the crush and synapses between these axons and the M-axon evoked EMGs caudal to the wound. HRP backfills caudal to a crush 8 days postoperatively showed that one ascending fiber was spared (Zottoli and Freemer, 2003). Larger trunk EMG responses were recorded in 4 of 18 axons (range = 0.107–0.386 mV), although these values were well below the smallest EMGs recorded in control fish (i.e., 0.7 mV). EMG responses elicited by M-axon activation recorded prior to a selective axotomy were abolished post-axotomy which indicates that they result from M-axon sprouts synapsing on appropriate post-synaptic targets (Figure 8).

The small amplitude EMGs recorded from SML-crush fish elicited by stimulation of the Mauthner axon implies that the resultant muscle contraction would not be sufficient to cause recovered startle responses. In support of this conclusion, trunk EMG responses during a post-injury startle response in a free-swimming fish were not due to M-cell activation (Figure 10).

### Mauthner-Cells and Their Sprouts Survive for Long Postoperative Intervals

Mauthner-cell death has been reported after SML-transection at 15.6°C; three of four cells in two fish were atrophied and one cell was missing 421 days (∼1.2 years) postoperatively (Figure 12 in Zottoli et al., 1984). In this study, cell death of one of a pair of M-cells did occur 434 days (∼1.2 years) after an SML-crush (Figure 9). Four fish studied for EMG responses 198–214 postoperatively had M-cell somata with substantial Nissl substance and none of the cells were chromatolyzed or swollen. In addition, axons filled with Lucifer yellow maintained extensive sprouting for at least 434 days postoperatively. Some somata appeared somewhat shrunken and, as a result, we cannot eliminate the possibility that the cells might atrophy and die at longer postoperative intervals.

A subgroup of larval lamprey reticulospinal neurons that include the M-cell have limited regenerative capacity (Davis and McClellan, 1994; Jacobs et al., 1997; Zhang et al., 2005). As a result, they have been classified as “bad regenerating” neurons (Davis and McClellan, 1994; reviewed in Rodemer et al., 2020). For example, M-axons of larval lamprey send sprouts between 2.5 and 5 mm past a whole cord transection in less than 10% of the axons studied (Jacobs et al., 1997; Sobrido-Cameán et al., 2019). Fluoro-Jade staining that labels degenerating neurons, TUNEL-positive labeling that marks cells undergoing apoptosis, and a complete loss of Nissl staining, are correlated with cells that are considered “bad regenerators.” Thus, M-cells are unlikely to survive axotomy (Shifman et al., 2008; Busch and Morgan, 2012). In contrast, our results demonstrate that adult goldfish M-cells maintain functional supraspinal connections and M-axon sprouts over long postoperative intervals.

### The Presence of the Mauthner-Cell Is Not Necessary for Post-injury Startle Responses

If M-cell regrowth does not contribute to post-injury startle responses, then removal of the cells should not influence the emergence of this behavior. In fact, fish that have had M-cell ablation followed by SML-crush (ablation-crush) display post-injury startle responses that are not significantly different in frequency, latency, and kinematic parameters as compared to post-injury startle responses after SML-crush alone.

The neuronal circuitry responsible for post-injury startle responses has not been identified. Non-M-cells are known to initiate startle responses in adult goldfish in the absence of the M-cell. Lesions that remove the M-cell and its initial segment (Eaton et al., 1982; Nissanov and Eaton, 1989) or cell-specific ablation (Zottoli et al., 1999) of M-cells does not abolish startle responses evoked by abrupt, acoustic/vibratory stimulation. These non-M-cell startle responses typically have longer latencies than those evoked by M-cells but have similar mechanical performance as compared to M-cell responses of control fish (Eaton et al., 1982, 2001; Zottoli et al., 1999). In contrast, kinematic parameters of post-injury startle responses differ from those of controls in this study and that of Zottoli and Freemer (2003). Thus, the emergent behavior after SML-crush may not involve parallel pathways revealed by M-cell ablation. Studies are needed to test whether parallel startle circuits formed by M-cell morphological homologs found in segments 5 and 6 of the medulla oblongata (Nakayama and Oda, 2004) may be part of the regenerative circuitry.

Our experimental approach is limited to “snapshots” at particular postoperative intervals, and, as a result, we cannot tell if M-axon sprouts are constantly remodeling or stable over time. M-axon sprouts that choose aberrant pathways may form synapses that stabilize the sprout and prevent further regrowth thus preventing contributions of the M-cell to post-injury startle responses. A Teflon barrier placed between rostral and caudal stumps of a transected goldfish spinal cord resulted in “arrested” axonal regrowth even after removal of the Teflon (Bernstein and Bernstein, 1967). The arrested growth has been hypothesized to result from synapse formation rostral to the Teflon block. The putative re-establishment of synapses by the M-cell may result in “contact inhibition” and a cessation of growth (Bernstein and Bernstein, 1967, 1969). Alternatively, the extensive sprouting of M-axons, albeit in aberrant pathways, may reach a neuronal volume that may limit further growth, as conceptualized by “the principle of conservation of total axonal arborization” (Devor and Schneider, 1975; Wood and Cohen, 1981; Sabel and Schneider, 1988). These possible mechanisms are not mutually exclusive.

## Conclusion

Axotomized M-cells of adult goldfish maintain supraspinal connections, display extensive, aberrant sprouting, elicit small trunk EMG responses, and survive for long postoperative intervals despite little or no activation of trunk or tail musculature caudal to the wound. The pathway choice of adult M-axon sprouts suggests an inability to recognize pathways taken during development and/or a redirection by the presence of inhibitory molecules (Ghosh and Hui, 2018; Sobrido-Cameán et al., 2019) that limits the ability of the M-cell to participate in post-injury startle responses.

The dynamic nature of sprouting, pathway choice, restructuring, and synapse formation cannot be easily revealed by static “snapshots.” The continued development of imaging techniques that allow continuous sampling is necessary to answer why some cells are limited in their ability to functionally regenerate (Bhatt et al., 2004; Kerschensteiner et al., 2005; Zhang et al., 2005; Dray et al., 2009; Laskowski and Bradke, 2013; Hu et al., 2018; Hecker et al., 2020b).

## Data Availability Statement

The raw data supporting the conclusions of this article will be made available by the authors, without undue reservation.

## Ethics Statement

The animal study was reviewed and approved by Williams College IACUC.

## Author Contributions

SZ and DF conceived and designed the experiments and wrote the manuscript. SZ, SN, AD, and JH performed the experiments and analyzed the data. All authors contributed to the article and approved the submitted version.

## Conflict of Interest

The authors declare that the research was conducted in the absence of any commercial or financial relationships that could be construed as a potential conflict of interest.

## Publisher’s Note

All claims expressed in this article are solely those of the authors and do not necessarily represent those of their affiliated organizations, or those of the publisher, the editors and the reviewers. Any product that may be evaluated in this article, or claim that may be made by its manufacturer, is not guaranteed or endorsed by the publisher.
